# A modified cementing technique using BoneSource to augment fixation of the acetabulum in a sheep model

**DOI:** 10.3109/17453674.2010.501740

**Published:** 2010-07-16

**Authors:** A John Timperley, Iulian Nusem, Kathy Wilson, Sarah L Whitehouse, Pieter Buma, Ross W Crawford

**Affiliations:** ^1^Princess Elizabeth Orthopaedic Centre, Royal Devon and Exeter Hospital, ExeterUK; ^2^Logan Hospital, Logan City; ^3^Medical Engineering Research Facility; ^4^Institute of Health and Biomedical Innovation, Queensland University of Technology, The Prince Charles Hospital, Brisbane, QueenslandAustralia; ^5^Department of Orthopaedics, Radboud University Nijmegen Medical Centre, NijmegenThe Netherlands

## Abstract

**Background and purpose:**

Our aim was to assess in an animal model whether the use of HA paste at the cement-bone interface in the acetabulum improves fixation. We examined, in sheep, the effect of interposing a layer of hydroxyapatite cement around the periphery of a polyethylene socket prior to fixing it using polymethylmethacrylate (PMMA).

**Methods:**

We performed a randomized study involving 22 sheep that had BoneSource hydroxyapatite material applied to the surface of the acetabulum before cementing a polyethylene cup at arthroplasty. We studied the gross radiographic appearance of the implant-bone interface and the histological appearance at the interface.

**Results:**

There were more radiolucencies evident in the control group. Histologically, only sheep randomized into the BoneSource group exhibited a fully osseointegrated interface. Use of the hydroxyapatite material did not give any detrimental effects. In some cases, the material appeared to have been fully resorbed. When the material was evident in histological sections, it was incorporated into an osseointegrated interface. There was no giant cell reaction present. There was no evidence of migration of BoneSource to the articulation.

**Interpretation:**

The application of HA material prior to cementation of a socket produced an improved interface. The technique may be useful in humans, to extend the longevity of the cemented implant by protecting the socket interface from the effect of hydrodynamic fluid flow and particulate debris.

## Introduction

Cemented ultra-high molecular weight polyethylene sockets remain the gold standard for use in hip replacement surgery in the elderly patient (Levy et al. 2000). However, the survivorship of these implants has been reported to fall at an increasing rate in the second decade after implantation. In addition, the results using cemented sockets in younger patients are often inferior even in the short term (Kobayashi et al. 1997). It has been suggested that late aseptic loosening of cemented acetabular components is governed by the progressive, 3-dimensional resorption of the bone immediately adjacent to the cement mantle (Schmalzried et al. 1992). It has been suggested that progressive loosening is initiated by the ingress of fluid and later debris, by hydrodynamic forces acting around the periphery of the socket (Schmalzried et al. 1992). This process begins circumferentially at the intraarticular margin and progresses towards the dome of the implant. This theory is supported by the frequent appearance of a radiolucent line at the edge of DeLee Charnley zone 1 that tends to extend with time around the interface. While it is possible to achieve good penetration of trabecular bone and sound mechanical fixation in the central portion of the acetabulum, it can prove difficult to achieve good mechanical interlock at the periphery, since this bone can be sclerotic and thin.

Hydroxyapatite (HA) has previously been used as a coating on total hip prostheses because it is capable of inducing new bone growth over a gap and enhances stability of an implant (Söballe et al. 1993). HA granules could be of use in achieving osseointegration of polymethylmethacrylate (PMMA) cement against bone at the periphery of the socket and in preventing fluid ingress and the advance of particulate debris. An “interface bioactive bone cement technique” has previously been advocated for use in both the knee and the hip, in an attempt to improve the results when cement is used for fixation (Oonishi et al. 2001a). BoneSource powder (Stryker Leibinger, Mahwah, NJ) consists of a mixture of anhydrous di-calcium phosphate (DCPA) and tetracalcium phosphate (TTCP), which are blended together in a certain ratio. When this powder mixture is mixed with the sodium phosphate solution provided, the resulting reaction produces HA as a crystalline phase in the set cement.

**Figure 1.**
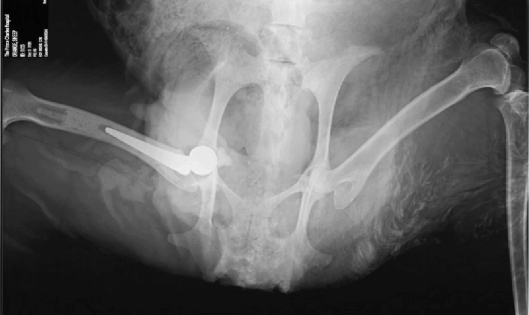
Sheep pelvis with hip replacement in situ.

Sheep and goats have often been used in studies involving hip arthroplasty. Instrumented endoprostheses in both animals have shown load orientation similar to those reported in humans, and maximum joint forces up to 110% of body weight have been recorded (Bergmann et al. 1984). Both models are considered to be suitable for studies of this type. Other authors have confirmed sheep to be an appropriate model for hip arthroplasty (Phillips et al. 1987, Brumby et al. 1998, Barker et al. 2000). The bony acetabulum of the sheep is made up of the 3 bones of the pelvis, as it is in man. However, there is little cancellous bone within the ovine socket. The walls of the socket are thin, making it very difficult to expose cancellous bone at the rim of the acetabulum. This means that the ovine acetabulum anatomy is an ideal model to test the efficacy of bioactive material against sclerotic bone at the rim (Figure 1). In the clinical situation, this is the most important area to achieve micro-interlock and thereby prevent the ingress of fluid and debris. The anatomy of the soft tissue around the ovine hip is very similar to the arrangement in humans.

We examined, in sheep, the effect of interposing a layer of hydroxyapatite cement around the periphery of the socket before fixing a polyethylene socket using polymethylmethacrylate (PMMA). The nature of the implant-bone interface was established by radiographic and histological means.

## Material and methods

22 sheep were randomly allocated to 2 groups using sealed envelopes, after initial preparation of the socket. In one group, BoneSource hydroxyapatite (HA) material was applied to the rim of the socket after preparation of the bony surface. In both groups, a polyethylene socket (Stryker Orthopedics, Mahwah, NJ) was then fixed into the acetabulum using a standardized cementing technique described below. Ethics committee approval for this project was obtained from the animal ethics committee of Queensland University of Technology in 2002.

The all-polyethylene cups that were inserted had a hemispherical geometry with a 32-mm external diameter and a 22-mm internal diameter. The femoral component was of the Exeter type (Stryker), and was specially made for implantation into animals. Simplex (PMMA) bone cement (Stryker) was used to fix both components.

BoneSource consists of a kit containing a syringe of 0.25 mL sodium phosphate mixing solution and 10 g hydroxyapatite cement powder. The solution and powder are mixed together in a mixing bowl using a spatula until thoroughly blended and then introduced into a 10-mL syringe. Within 4 h, BoneSource will mainly convert into hydroxyapatite, at which point it becomes insoluble.

### Perioperative protocols

The animals were fasted overnight and the area over the right jugular vein was clipped, the vessel cannulated, and pre-medication and analgesia administered 15 min before surgery by intravenous injection of Buprenorphine (0.01 mg/kg). Anesthesia was induced with Propofol and endotracheal intubation was performed. Anesthesia was maintained by Propofol infusion. All animals were given prophylactic antibiotics intravenously: Cephalothin Sodium (1 g), Tribactral (Trimethoprim 40 mg/mL and Sulfadoxine 200 mg/mL), and Gentamicin (1 mg/kg) intramuscularly.

Using a posterior approach to the hip, the rim of the acetabulum was exposed and the acetabular labrum excised. Three 6-mm drill holes were then made, 1 each into the 3 main bones of the pelvis. 2 reamers of increasing size were employed on a powered tool to remove cartilage and (wherever possible) the subchondral bone. In most areas it was not possible to expose cancellous bone, except in the drill holes. A trial socket was inserted to check that the cavity was large enough to accommodate an implant. The walls of the socket were usually thin following reaming, and if a perforation was noted this was filled with autogenous bone graft from the reamings or with BoneSouce. Multiple 2-mm drill holes were then made around the rim of the socket using the stepped drill, taking care not to perforate the inner table of bone. The bone was then thoroughly lavaged with a pressurized system to clean the interstices of the exposed bone and to clean away any blood. The bone was kept as dry as possible for the socket fixation by the use of hydrogen peroxide-soaked swabs.

At this point, the technique differed depending on whether or not the animal had been randomized to have the modified surgical technique using BoneSource. For the BoneSource group, the material was mixed with 10 mL sodium phosphate to a consistency that could just be injected through the end of a 10-mm syringe. A continuous thin layer of the material was squeezed out of the nozzle, around the rim of the acetabulum and onto the bone of the true floor of the socket. A spatula was then used to smear the material to a limit approximately 25 mm inside the socket edge. A single mix of Simplex was also prepared to allow introduction into the socket 3.5 min after the start of mixing. This timing was planned to coincide with completion of the BoneSource smearing technique.

**Figure 2.**
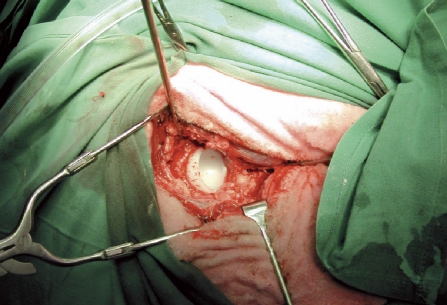
Operative exposure.

In all animals, the bone cement was introduced digitally into the socket at 3.5 min and a rubber pressurizer was used to seal against the rim of the socket, allowing pressure to be exerted on top of the cement in the socket. 6 min after the start of mixing, the pressurizer was removed and the socket was introduced by hand into the cement in a position anatomically appropriate for the orientation of the ovine acetabulum. Excess cement and any exuded BoneSource, if present, was cleaned from around the rim of the implanted socket (Figure 2).

The femoral component was then cemented into the prepared femur and the hip was reduced. Closure of the external rotators was not usually possible. Fascia and skin were closed. Marcain was injected into the edges of the wound.

### Postoperative treatment

The animal was extubated and recovered on its side. Temgesic (0.01 mg/kg) was administered by intramuscular injection for 24 h following surgery. The animal was allowed to mobilize freely and most sheep were partially weight bearing on the limb within a week of surgery. After 2 days in individual housing, the animals were transferred to group housing in the animal house.

The animals were killed 6 months after implantation. They were given tetracycline 7 days before, and calcein 1 day before the explant surgery. The hips were removed under anesthesia and immediately radiographed, and then fixed in formalin. Tissue was sent for culture and antibiotic sensitivity testing when infection was suspected clinically.

### Radiographic evaluation

**Figure 3.**
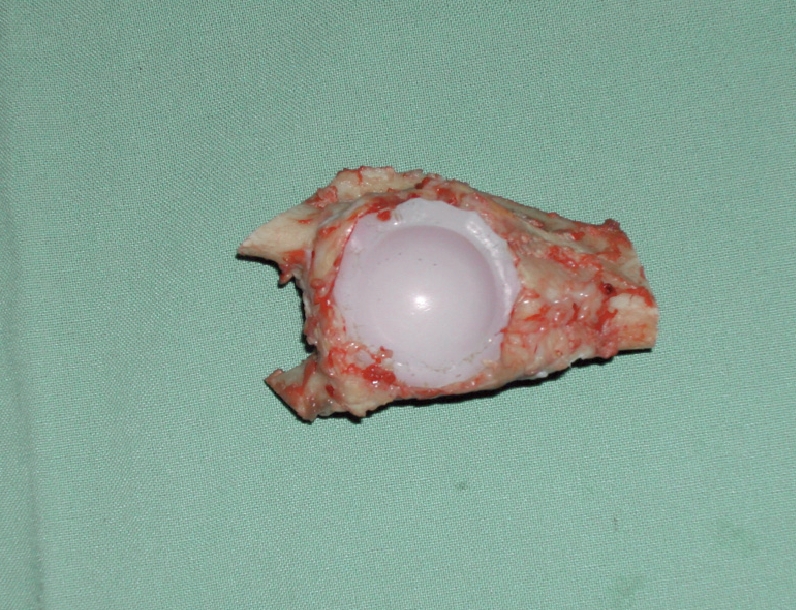
Harvested acetabulum with cup in place.

The acetabula were stripped of soft tissue attachments and removed following sectioning of the 3 bones of the pelvis (Figure 3). Each specimen was radiographed “en face” and at 90° to the face of the acetabular opening. Radiolucencies around the implanted material were noted and if present around the entire interface, the socket was described as demarcated.

### Histological examination

The specimens were sectioned from 12 o'clock to 6 o'clock and viewed “en face” with the transverse ligament positioned inferiorly. Sections 3 mm thick were prepared and 3 sections then stained with hematoxylin. Undecalcified specimens were also prepared for analysis.

The hematoxylin specimens were assessed by light microscopy and the nature of the interfaces established. Evidence of abnormal biological activity and remodeling was noted along with the presence or absence of BoneSource material in the sections. Polarized light was used to inspect the interface tissues for polyethylene debris. The articulating surfaces of the explanted sockets were inspected by light microscopy (by PB) for evidence of abnormal wear or HA material embedded in the articular surface.

The thickness of the interface between host bone and man-made material was measured after digitization of the radiographs at 250× magnification. For purposes of analysis, the sections were divided into 3 zones corresponding to DeLee and Charnley (1976) and an average of 3 measurements made in each zone was calculated for each of the 3 sections prepared from the animals.

### Statistics

Following normality testing, non-parametric methods were used to compare continuous data (Mann-Whitney U-test). Frequencies were compared using chi-squared or Fisher's exact test as appropriate. The primary endpoint was osseointegration at the interface, which was defined as having no soft tissue at any point in the interface examined. This is using Brånemark's definition (Albrektsson et al. 1981) of osseointegration as “a direct—at the light microscopic level—contact between living bone and implant”. Secondary endpoints were the presence of radiolucent lines, histological examination, and interface thickness. Multiple testing for this level of testing was adjusted for using Bonferroni's correction. The significance level for these tests then becomes p = 0.017.

## Results

Of the 22 sheep, 11 were randomly allocated into each group. There were 4 deep infections, 3 in the BoneSource group. In all 4 cases, the sockets were obviously macroscopically loose on explantation and there was a thick fibrous interface between acrylic bone cement and host bone. Histologically, polymorphonuclear leukocytes were seen in clusters at the cement-bone interface. In each of the 3 cases in which hydroxyapatite material had been implanted, it was found to have been fully resorbed. These 4 infected cases were excluded from further radiological or histological examination.

### Radiographic appearance

After exclusion of the infected cases, there were 18 sets of radiographs available for inspection: 8 in the BoneSource group and 10 in the PMMA-only group. Only 1 out of 8 of the BoneSource group was fully demarcated, as compared to 9 out of 10 in the control group (implanted without HA) (p = 0.003). The number of DeLee and Charnley zones available for inspection was 24 in the BoneSource group and 30 in the control group. The control group exhibited more radiolucencies (after adjustment for multiple testing) than the BoneSource group: 27 of 30 (90%) and 6 of 24 (25%), respectively (p < 0.001 using the chi-squared test).

### Histological appearance

**Figure 4.**
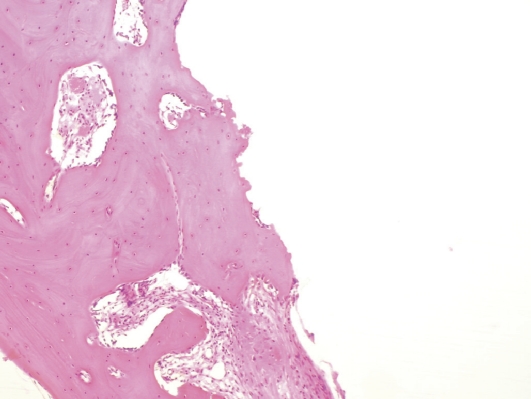
Osseointegrated interface with no BoneSource visible.

In addition to the infected cases, 3 further acetabula were excluded from histological assessment, as the sectioning of the specimen was inappropriate. There remained 7 cases in the BoneSource group and 8 controls with material available for histological assessment and measurement (Figure 4).

The histological appearances were described as “quiet” in 6 cases; in these, there was no increased bone turnover, no periosteal new bone or fibrocartilage formation, normal cellularity, and an absence of giant cells. 4 out of 7 of these were in the BoneSource group and 2 out of 8 were in the control group (Fisher's exact test, p = 0.3).

2 control cases had soft tissue extending around the whole of the interface, averaging 0.2 mm in thickness. None of the controls had an osseointegrated interface, as compared to 4 in the BoneSource group (Fishers exact test, p = 0.03).

We examined the average interface thickness. The data were not normally distributed; thus, non-parametric methods were used. The median interface thickness for the BoneSource group was 56 μm (IQR 870) and for the other group it was 805 μm (IQR 548) (Mann-Whitney U-test, p = 0.3).

Of the 7 BoneSource cases reviewed, the HA material was evident in histological sections from 4 sheep. In 1 case, there had been a technical problem at the time of surgery and PMMA and unresorbed HA had protruded into the pelvis. The other 3 cases were osseointegrated along almost the entire cement-bone interface, and small amounts of BoneSource were seen in direct apposition to—or encased in—trabecular bone. There were no adjacent giant cells and the histological picture was mature, with evidence of a well-fixed implant. The HA had been fully resorbed in the 3 other cases in which it had been used.

Examination with polarized light showed no evidence of polyethylene debris at the interfaces. In no case was embedded HA material identified in the articulation.

## Discussion

This study supports the results of a previous study using a rabbit model, in which hydroxyapatite was used at the cement-bone interface (Oonishi et al. 1989). The integrity of the bone cement interface was shown to be improved when hydroxyapatite granules were interposed at the interface (Oonishi et al. 1989, Oonishi 1991). Based on this experimental result in animals, HA granules were smeared onto the bone-cement interface before cement fixation in a group of patients (Oonishi et al. 2001b). At surgery, HA granules (2–5 g) were smeared manually over the bone surface just before cement fixation of the acetabular components. There were no untoward clinical complications attributable to the use of HA granules, as reported after a mean follow-up time of 10 years. The study group comprised 218 hips with full data available. The overall loosening rate was 3% for the acetabular component.

In our sheep model, only cases treated with BoneSource showed evidence of complete osseointegration at the cement-bone interface. We did not observe the radio-dense line at the interface reported by Oonishi et al. However, with our model, only the periphery of the PMMA mantle would have been adjacent to HA paste and it would have been difficult to see this change in density if it occurred.

Oonishi et al. maintain that using the interface bioactive bone cement technique (IBBC), the crystalline HA is unresorbable. However, other authors have reported HA coatings on implants undergoing resorption (Lintner et al. 1994, Overgaard et al. 1998, Tonino et al. 2001), and we noted that the BoneSource was resorbed in many cases in our animal model.

Oonishi et al. (2000) confirmed that there were histologically negligible foreign body reactions in the specimens retrieved after ten years of service in vivo. They attributed this to the fact that the particle size of HA granules was relatively large (100–300 μm) and that most of the granules were incorporated. Likewise, we did not observe giant cells at the interface in any of the animals, and the histological appearance adjacent to the BoneSource material was quiet in all cases where there was unresorbed material. It appears that the size of the HA granules is not the most important determinant of an adverse histological picture.

Although there is no doubt that a surface coating of HA can enhance the stability of an implant, it is also true that the use of HA in no way substitutes for stable mechanical fixation (Munting and Verhelpen 1995, Lai et al. 2002). Whereas Oonishi et al. used HA material as a thick layer over the whole interface to enhance fixation, we only used a coating of the material around the rim of the socket. Stable mechanical fixation can be achieved in the trabecular bone of the socket, but interlock is not achievable between PMMA and a sclerotic bony surface. Our aim was to enhance fixation at the periphery and thereby to possibly protect the rest of the socket interface from the effect of hydrodynamic fluid flow and particulate debris (Schmalzried et al. 1992). The material appears to achieve this goal, and the next step would be to formally try the technique in humans in the hope that long-term benefit from its use will become apparent.
